# The taxlist package: managing plant taxonomic lists in R

**DOI:** 10.3897/BDJ.6.e23635

**Published:** 2018-05-02

**Authors:** Miguel Alvarez, Federico Luebert

**Affiliations:** 1 Universität Bonn, Bonn, Germany

**Keywords:** ecoinformatics, database, taxon concept, taxon view, Turboveg, vegtable

## Abstract

Taxonomic lists are crucial elements of vegetation-plot databases and provide the links between original entries, reference taxon views and different taxon concepts. We introduce the R package *taxlist* in the context of object-oriented modelling for taxonomic lists. This package provides a data structure based on species lists in Turboveg, which is a software broadly used for the storage of vegetation-plot databases and implements functions for importing and handling them prior to statistical analysis. We also present a schema for relational databases, compatible with *taxlist* objects and recommend its use for handling diversity records.

## Introduction

Vegetation-plot databases are increasingly gaining importance, not only as a way to host historical vegetation data or to store data collected in ongoing research projects, but also for storing vegetation-plot observations including types of syntaxonomical classifications in the context of the Braun-Blanquet approach (Dengler et al. 2011). Several software applications are suitable for storing and handling this kind of database. For instance, Turboveg (Hennekens and Schaminée 2001) is one of the most widespread software used for storage of vegetation-plot databases and for data sharing in Europe (Dengler et al. 2008, Schaminée et al. 2009) and was therefore recognised in 1994 as the official software for storing vegetation data by the Working Group Council of the European Vegetation Survey (Hennekens and Schaminée 2001).

Taxonomic lists (i.e. species lists) are crucial components of vegetation-plot databases and several authors have raised concerns about the consequences of inconsistent nomenclatorial applications in downstream statistical analyses (Ewald 2001, Jansen and Dengler 2010, Wiser et al. 2011). Many applications attempt to standardise nomenclatures comparing custom species lists with on-line databases. Many of those applications are available in R (R Core Team 2017). Some examples are the packages *Taxonstand* (Cayuela et al. 2012) that compares lists with the Taxonomic Resolution Services (e.g. Tropicos and PLANTS Database), *taxize* (Chamberlain and Szöcs 2013) which uses the same sources as *Taxonstand* but some additional ones (e.g. ITIS and IPNI) and *vegdata* (Jansen and Dengler 2010) which imports tables from Turboveg databases as well as data from the German database "VegetWeb", providing some functions for handling data previous to statistical analysis. These packages assume that retrieved accepted taxonomic names replace (overwrite) the names from the input data according to a standard synonymy. However, a universal consensus on the delimitations of a taxon is frequently not achieved and taxonomy is either a static discipline (Koperski et al. 2000, Jansen and Dengler 2010, Cayuela et al. 2012, Holstein and Luebert 2017). When working with historical data, the links between old taxa names (see Table 1 for basic definitions) and the current accepted name have to be traceable, allowing tracking back inconsistencies in the data. This is also in agreement with Jansen and Dengler (2010), who remark that even uncertain information should be stored as in the source, since expert opinions or access to collected specimens, for instance, may allow the resolution of these uncertainties that otherwise will not be available for the statistical analysis.

Handling taxon concepts with their respective names requires relational structures rather than flat tables, as currently done by Turboveg and most of the mentioned R-applications. These structures may not only be useful for data storage but also for implementing consistent algorithms to manage the information contained in taxonomic lists, such as retrieving occurrence of taxa rather than names, building subsets by querying taxonomic relationships, quickly displaying diversity statistics and especially testing for consistency of the data.

While intensive discussions have been centred around data integration (e.g. Jones et al. 2006, Wiser et al. 2011, Wieczorek et al. 2012) and standardisation of taxonomic lists (e.g. Zhong et al. 1996, Jansen and Dengler 2010, Cayuela et al. 2012), applications integrating information modelling (data structure) and processes (functions) are required to facilitate information exchange amongst researchers and databases. Object-oriented modelling in the sense of Berendsohn (1997) is a way to integrate information and processes, which may be frequently required when sharing, changing and tuning taxonomic lists (Zhong et al. 1996). Thus, the object-oriented modelling of taxonomic lists has a huge potential for enhancing automatic procedures in sharing and merging information contained in vegetation data and also in other kinds of floristic, biological and even syntaxonomical records.

In this work, we aim to provide an information structure for storing taxonomic lists (the class *taxlist*) focusing on species lists contained in Turboveg databases. The main properties implemented in these objects are: 1) flexibility to include different degrees of available information (flat taxon lists, lists including taxon traits or hierarchical structures including taxon levels and parent-child relationships), 2) an automatic check of consistency of information contained in those objects (provided by validity checking), 3) quick display of information contained in the lists (summary methods) and 4) common processes implemented in specific functions.

## Taxlist objects and basic methods

The package *taxlist* can be installed from Comprehensive R Archive Network (CRAN). Alternatively it can also be installed from a GitHub repository (https://github.com/kamapu/taxlist) using the package *devtools*.

This package was programming using S4 (the fourth version of the programming language S), which is also implemented in R (Genolini 2008). S4 is an object-oriented programming language, where the object variables are called "slots" and the respective functions, "methods". S4 was selected for programming *taxlist* because the objects are formally defined, the implemented validity checking allows us to set "rules" for coherence in the content of taxonomic lists (e.g. no duplicated entries, relationships between taxa respecting hierarchy) and its capability for defining methods in accordance to the structure and content of taxonomic lists.

**Figure 1.**
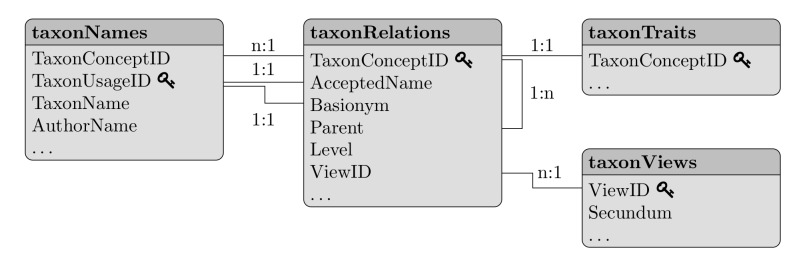
Relational model of information contained in objects of class *taxlist*. Each box is a column-oriented table including the name of the table (slot name in R) and the names of the mandatory columns. Lines indicate relationships between and within tables, while key symbols show the key fields in the respective tables. Dots suggest the possibility to extend the tables with custom columns. Single entries in table *taxonRelations* represent a taxon, while table *taxonNames* includes taxon usage names (accepted names and synonyms). Attributes of taxa (e.g. functional traits) are stored in table *taxonTraits* and table *taxonViews* includes sources determining the circumscription of a taxon, its accepted name and synonyms.

**Figure 2.**
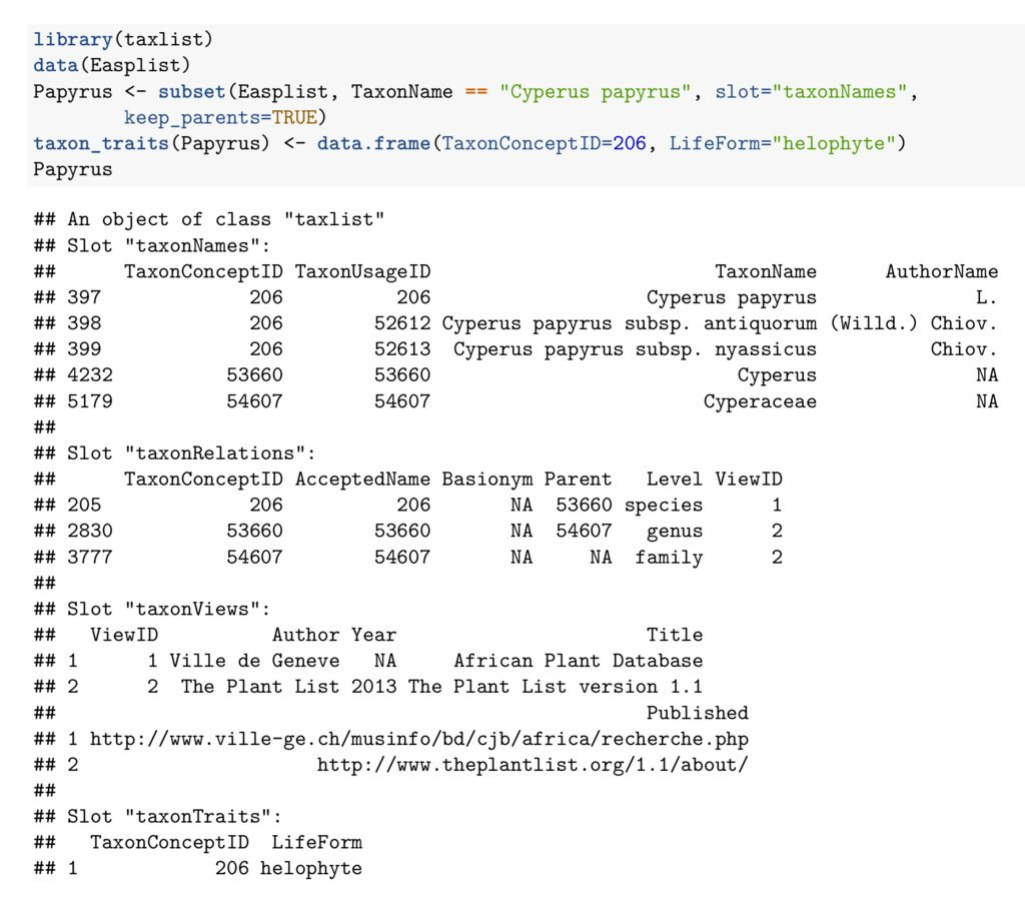
Taxon information structured in a taxlist object. The code loads the package and the installed example data "Easplist". Here the function *subset()* extracts the taxon *Cyperus papyrus* L. with its parents. Since the example does not have any information on taxon properties (traits), the life form for *C. papyrus* (identifier 206 in the example data) is inserted before to produce the print in the R console.

Each slot in *taxlist* objects is containing a column-oriented table (class *data.frame* in R). One of the most important features of the *taxlist* objects is the separation of taxon names from their relationships to taxon concepts (see Table 1 for definitions). Names and their associated information are stored in the slot *taxonNames* and linked to their respective concepts in the slot *taxonRelations* (Fig. 1). Single entries in slot *taxonRelations* are then considered as "potential taxa" (Berendsohn 1995) or "taxonyms" (Koperski et al. 2000). Following Zhong et al. (1996), the slot *taxonViews* contains the references that are used for taxon concepts. The slot *taxonTraits* contains attributes of the taxon concepts (e.g. functional traits, life form, conservation status, chorological classification etc.), that can be further used to produce the respective statistics (i.e. indicator figures, life form and chorological profiles; see also Fig. 2). This slot may content only a subset of the taxon concepts included in the *taxlist* object. The user is free to extend all those tables by adding new columns.

While the design of taxlist objects was inspired by the content of species lists in the software Turboveg (see also https://www.synbiosys.alterra.nl/turboveg), which are stored in DBF files, the main differences with *taxlist* are: 1) The content of slots *taxonNames* and *taxonConcepts* is stored in a single table called "species" in Turboveg and 2) taxon views and hierarchical structures are not explicitly supported in Turboveg. On the other hand, Turboveg also stores taxon attributes in a separated table called "ecodbase".

Empty objects are generated from a template or prototype by the function *new()*. Alternatively, they can be created from character strings containing accepted names or data frames with accepted names and synonyms by using the function *df2taxlist()*, which returns a taxlist object. In a similar way, it is possible to import species lists included in Turboveg databases through the function *tv2taxlist()*. In the latter case, the similar function *tax()* is available from the package *vegdata* (Jansen and Dengler 2010), but the output of *tax()* is a flat table of class *data.frame* and is not suitable for further processing in *taxlist*, except if they are further transformed by *df2taxlist()*.

Hierarchical taxonomic structures can be also implemented through parent-child relationships, which is an optional feature of *taxlist*. In that case, the information on taxonomic levels (i.e. names and hierarchical sequence) have to be set by the user (function *levels()*, see also Suppl. material 1).

Consistency of information in taxonomic lists is checked by the function *validObject()* in R. Validity checking in *taxlist* includes detecting occurrence of duplicated combinations (same taxon names with same author), duplicated identifiers (IDs) and orphaned entries, amongst others. In the special case of lists including taxonomic levels and parent-child relationships, it will be further checked that any parent entry is included as concept and that any child is at least one level lower than the respective parent.

The function *summary()* retrieves the number of names and taxon concepts included in the input object as well as the number of traits and references, occurrence of parent-child relationships and taxonomic levels. The function *summary()* can be also applied to single concepts indicated in the argument *ConceptID* that are queried either by ID numbers (integer value) or by names (character value). In that case, the respective overview displays the accepted name, taxon view, synonyms, taxonomic rank and parent concept in the console.

## Further methods

Several methods are provided to handle the information of *taxlist* objects; many of them allow adding, replacing and retrieving components of a taxonomic lists (e.g. *taxon_names()*, *taxon_relations()*, *taxon_traits()* and *taxon_views()*). The function *subset()* works as a query, building through logical operations or character matching. Using this function, a set of taxon concepts are extracted from an object, keeping the validity of output objects. The query is applied to the content of a slot (defined in argument *slot*), while children or parents of retrieved taxa can be preserved in the output (arguments *keep_children* and *keep_parents*). The function *clean* removes orphaned entries in order to recover the validity of objects affected by direct manipulation of slots.

The functions *add_concept()*, *add_synonym()*, *accepted_name()* and *change_concept()* are suitable to increment information contained in a taxlist object and to modify the relationships amongst taxon names. They facilitate common processes required for changing or tuning taxonomic classifications as proposed by Zhong et al. (1996). While these functions can be applied to modify databases, they can also be included in scripts used to prepare data for assessments, avoiding modifications of the source. All these functions contain restrictions to safeguard the validity of *taxlist* objects. For instance, moving names amongst concepts is only allowed for synonyms and a new accepted name should already be included as a synonym for the respective concept. Any movement of names across taxonomic concepts affects the link of a name to a taxon concept, but preserves the identity of the name including its authority.

Adding parent-child relationships amongst concepts (i.e. relatiohips amongsttaxa at different taxonomic ranks) imply the use of the function *add_parent()*, while the function *add_level()* should be used for including taxonomic ranks or even adding new levels into existing ranks (increase on taxonomic resolution).

Finally, the function *backup_object()* attempts to create backups of *taxlist* objects (and any object in an R session) as an R image stored in a zip-file for recovery purposes. The zip file will include, by default, a time stamp (the date of backup) and a suffix in the case of more than one backup produced in the same day. Backup files can be loaded to a session with the function *load()*, while the function *load_last()* will automatically select the last created backup within a folder.

The use of the above mentioned functions is demonstrated in Suppl. material 1, which is also included as vignette in the package *taxlist*, as well as in the respective documentation.

## Final remarks and outlook

Since the definition of *taxlist* as a class in R enables its use as a component in other S4 objects, for example as a slot, it can be implemented in any newly defined class connecting diversity records with taxonomic data. This capability is demonstrated with the package *vegtable* (Alvarez 2017), which handles data from vegetation plots databases in R, including the homonymous S4 class. In *vegtable* objects, a slot *species* contains the list of taxa and is restricted to *taxlist* objects, while the respective taxon usage names are linked to records stored in the slot samples.

While a series of packages has been implemented in R for handling information of species and taxonomic lists, the implementation of a structured object class and respective functions in the framework of object-oriented programming is a novel feature in *taxlist*. This package is suitable for formatting floristic lists from raw data and testing the consistency of the respective information previous to its storage in a relational database, but also to make modifications of data through an R script previous to its assessment without the necessity of modifying the data source.

A combined work of *taxlist* with packages dealing with standardisation of nomenclature is demonstrated by the implementation of the function *tnrs* from the package *taxize* (Chamberlain and Szöcs 2013). Further alternatives are the packages *vegdata* (Jansen and Dengler 2010) and *Taxonstand* (Cayuela et al. 2012). All those packages will provide functionalities regarding equalisation of taxonomic nomenclatures, while *taxlist* will keep the relations between original entries and the reference nomenclature (a taxonomic view) and provide functions for further handling of data.

## Supplementary Material

Supplementary material 1 Applying taxlist to species lists on diversity records Data type: R vignette
File: oo_178681.html Alvarez, M

## Figures and Tables

**Table 1.** List of terms as used in the taxlist package.

**Accepted name**: The name used for designating a taxon. According to the International Code of Botanical Nomenclature (McNeill et al. 2012), its description could be vouchered by a type, while its taxonomic circumscription may vary according to different taxon views (Koperski et al. 2000, Jansen and Dengler 2010).
**Combination**: The name of a taxon at the species level or below, which includes the name of the genus and further epithets (McNeill et al. 2012). A combination should also indicate the respective author for differentiation amongst homonyms.
**Potential taxon**: Proposed by Berendsohn (1995) as a technical solution for taxon- and name-based databases in cases where the same taxon name could have different taxonomic circumscriptions (e.g. when a taxon gets split or when divergent views are applied in the databases). The potential taxon indicates the taxonomic circumscription of an accepted name according to a reference (taxon view). Thus, the proper way to designate a potential taxon is by the concatenation of 1) accepted name, 2) author of the name and 3) reference.
**Synonym**: A name applied to a taxon, alternative and subordinated to its accepted name. Synonyms are subdivided into homotypic or nomenclatural when they share the same typus as the accepted name and heterotypic or taxonomic when they are described based on different types (McNeill et al. 2012).
**Taxon**: A taxonomical entity belonging to any rank of the taxonomic classification.
**Taxon concept**: Also called "taxonomic concept", it refers to the taxonomic circumscription denoted by a name according to an "opinion" (taxon view). It is fairly applied as the synonym of "potential taxon" and "taxonym" (Berendsohn 1995).
**Taxon name usage**: Application of a name to design a taxon concept, regardless of its status as accepted name or synonym. This term refers to alternative plant names also in the package *vegdata* (Jansen and Dengler 2010) and in the "Veg-X" framework (Wiser et al. 2011).
**Taxon view**: The reference used for determining hierarchical position and circumscription of a taxon concept. This term was introduced by Zhong et al. (1996) to make visible changes in the circumscription of a taxon concept that has preserved its accepted name or to raiseawareness about taxonomic discrepancies amongst references.
**Taxonym**: This term was proposed by Koperski et al. (2000) as the German synonym for "potential taxon" (Jansen and Dengler 2010).
**Syntaxon**: An abstract unit of phytocoenoses, which is defined by its species composition and plant-sociological patterns (co-occurrence). As in the case of a taxon, a syntaxon is incorporated into a hierarchical system (Weber et al. 2000).

## Hosting institution

University of Bonn, Germany.
