# Supplementary material for: The taxlist package: managing plant taxonomic lists in R
Source: Biodivers Data J. 2018 May 2;(6):e23635. doi: 10.3897/BDJ.6.e23635 (PMC5943443; doi:10.3897/BDJ.6.e23635)
Supplement: Supplementary material 1 — Applying taxlist to species lists on diversity records [file bdj-06-e23635-s001.html]

Applying taxlist to species lists on diversity records


# Applying taxlist to species lists on diversity records

#### *Miguel Alvarez*

#### *2018-01-05*

# 1. Getting started

The package `taxlist` aims to implement an object class and functions (methods) for handling taxonomic data i **R**. The homonymous object class `taxlist` can be further linked to biodiversity records (e.g. for observations in vegetation plots).

The `taxlist` package is developed on the repository **GitHub** (https://github.com/kamapu/taxlist) and can be installed in your R-session using the package `devtools`:

```
library(devtools)
install_github("kamapu/taxlist", build_vignettes=TRUE)
```

Since this package is already available in the Comprehensive R Archive Network (CRAN), it is also possible to install it using the function `install.packages`:

```
install.packages("taxlist", dependencies=TRUE)
```

Of course, you have to load `taxlist` into your R-session.

```
library(taxlist)
```

For accessing to this vignette, use following command:

```
vignette("taxlist-intro")
```

# 2. Extracting a species list from a vegetation table

## 2.1 Example data

One of the main tasks of `taxlist` is to structure taxonomic information for a further linkage to biodiversity records. This structure have to be on the one side consistent with taxonomic issues (e.g. synonyms, hierarchies, etc.), on the other side have to be flexible for containing different depth of information availability (from plain species lists to hierarchical structures).

In this guide, we will work with a species list from phytosociological relevés collected at the borderline between the **Democratic Republic of the Congo** and **Rwanda** (Mullenders 1953 *Vegetatio* 4(2): 73–83).

The digitized data can be loaded by following command:

```
load(url("https://github.com/kamapu/thisdataismine/raw/master/data/Cross.rda"))
```

The data is formatted as `data.frame` in **R**, including the names of the species in the first column:

```
head(Cross[,1:8])
```

```
##                 TaxonName 3094 3093 3092 3095 3096 3097 3098
## 1   Eragrostis tenuifolia    + <NA> <NA> <NA> <NA> <NA> <NA>
## 2        Cyperus sublimis <NA>    + <NA> <NA> <NA> <NA> <NA>
## 3    Digitaria abyssinica    +    1    2    2    2    3    1
## 4 Hyparrhenia filipendula <NA> <NA> <NA> <NA> <NA> <NA> <NA>
## 5    Erigeron floribundus    +    1 <NA> <NA> <NA> <NA> <NA>
## 6            Aerva lanata    +    1 <NA> <NA> <NA> <NA> <NA>
```

## 2.2 From plain list to taxlist

As already mentioned, the first column in the cross table contains the names of the species occurring in the observed plots. Thus, we can use this character vector to construct a `taxlist` object. This can be achieved through the function `df2taxlist`.

```
Splist <- Cross[,"TaxonName"]
Splist <- df2taxlist(Splist)
summary(Splist)
```

```
## object size: 8.5 Kb 
## validation of 'taxlist' object: TRUE 
## 
## number of names: 35 
## number of concepts: 35 
## trait entries: 0 
## number of trait variables: 0 
## reference entries: 0
```

Note that the function `summary` provides a quick overview in the content of the resulting object. This function can be also applied to a specific taxon:

```
summary(Splist, "Erigeron floribundus")
```

```
## ------------------------------ 
## concept ID: 5 
## view ID: none 
## level: none 
## parent: none 
## 
## # accepted name: 
## 5 Erigeron floribundus  
## ------------------------------
```

## 2.3 Resolving taxonomic names with taxize

One of the main concerns is to resolve nomenclatorial issues, especially when working with historical data or combining data from different sources. For this purpose, `taxlist` implements a method for the function `tnrs` (package `taxize`):

```
summary(Splist)
```

```
## object size: 8.5 Kb 
## validation of 'taxlist' object: TRUE 
## 
## number of names: 35 
## number of concepts: 35 
## trait entries: 0 
## number of trait variables: 0 
## reference entries: 0
```

```
Splist <- taxlist::tnrs(Splist)
summary(Splist)
```

```
## object size: 14.6 Kb 
## validation of 'taxlist' object: TRUE 
## 
## number of names: 45 
## number of concepts: 35 
## trait entries: 0 
## number of trait variables: 0 
## reference entries: 0
```

While after this procedure, the number of taxa remained as 35, the number of taxon names increased, indicating that some of the names in the original source are currently considered as synonyms. This is for example the case of *Erigeron floribundus*:

```
summary(Splist, "Erigeron floribundus")
```

```
## ------------------------------ 
## concept ID: 5 
## view ID: none 
## level: none 
## parent: none 
## 
## # accepted name: 
## 36 Conyza sumatrensis var. leiotheca (S.F. Blake) Pruski & G. Sancho 
## 
## # synonyms (1): 
## 5 Erigeron floribundus  
## ------------------------------
```

At this point it is important to mention that while most resolvers are offering ways to replace names by accepted ones, in `taxlist` objects both, accepted names and synonyms are preserved. Only typographical errors and names of authors will be changed in the object. This is important in the compilation of diversity records (i.e. vegetation plot databases), where the stored data have to be faithful to the source. Thus data connected to a taxon name can be automatically assigned to the correspondent taxon concept without to modify the record’s entry. An overview to the accepted names and possible changes can be displayed as follows:

```
accepted_name(Splist)[1:5,]
```

```
##   TaxonConceptID TaxonUsageID                         TaxonName
## 1              1            1             Eragrostis tenuifolia
## 2              2            2                  Cyperus sublimis
## 3              3            3              Digitaria abyssinica
## 4              4            4           Hyparrhenia filipendula
## 5              5           36 Conyza sumatrensis var. leiotheca
##                        AuthorName
## 1    (A. Rich.) Hochst. ex Steud.
## 2             (C.B. Clarke) Dandy
## 3     (Hochst. ex A. Rich.) Stapf
## 4                 (Hochst.) Stapf
## 5 (S.F. Blake) Pruski & G. Sancho
```

# 3. Built-in data set

## 3.1 Easplist

The installation of `taxlist` includes the data `Easplist`, which is formatted as a `taxlist` object. This data is a subset of the species list used by the database **SWEA-Dataveg** (GIVD ID AF-006):

```
data(Easplist)
summary(Easplist)
```

```
## object size: 739.8 Kb 
## validation of 'taxlist' object: TRUE 
## 
## number of names: 5337 
## number of concepts: 3935 
## trait entries: 51 
## number of trait variables: 1 
## reference entries: 2 
## 
## concepts with parents: 3745 
## concepts with children: 1214 
## 
## hierarchical levels: form < variety < subspecies < species < genus < family 
## number of concepts in level form: 1
## number of concepts in level variety: 110
## number of concepts in level subspecies: 81
## number of concepts in level species: 2528
## number of concepts in level genus: 1025
## number of concepts in level family: 189
```

## 3.2 Access to slots

The common ways to access to the content of slots in `S4` objects are either using the function `slot(object, name)` or the symbol `@` (i.e. `object@name`). Additional functions, which are specific for `taxlist` objects are `taxon_names`, `taxon_relations`, `taxon_traits` and `taxon_views` (see the help documentation).

Additionally, it is possible to use the methods `$` and `[` , the first for access to information in the slot `taxonTraits`, while the second can be also used for other slots in the object.

```
summary(as.factor(Easplist$LifeForm))
```

```
## Ch  H Hy  T 
##  5  7  3 36
```

## 3.3 Subsets

Methods for the function `subset` are also implemented in this package. Such subsets usually apply pattern matching (for character vectors) or logical operations and are analogous to query building in relational databases. The `subset` method can be apply to any slot by setting the value of the argument `slot`.

```
Papyrus <- subset(Easplist, grepl("papyrus", TaxonName), slot="names")
summary(Papyrus, "all")
```

Or the very same results:

```
Papyrus <- subset(Easplist, TaxonConceptID == 206, slot="relations")
summary(Papyrus, "all")
```

Similarly, you can look for a specific name.

```
Phraaus <- subset(Easplist, charmatch("Phragmites australis", TaxonName),
    slot="names")
summary(Phraaus, "all")
```

## 3.4 Hierarchical structure

Objects belonging to the class `taxlist` can optionally content parent-child relationships and taxonomic levels. Such information is also included in the data `Easplist`, as shown in the summary output.

```
summary(Easplist)
```

```
## object size: 739.8 Kb 
## validation of 'taxlist' object: TRUE 
## 
## number of names: 5337 
## number of concepts: 3935 
## trait entries: 51 
## number of trait variables: 1 
## reference entries: 2 
## 
## concepts with parents: 3745 
## concepts with children: 1214 
## 
## hierarchical levels: form < variety < subspecies < species < genus < family 
## number of concepts in level form: 1
## number of concepts in level variety: 110
## number of concepts in level subspecies: 81
## number of concepts in level species: 2528
## number of concepts in level genus: 1025
## number of concepts in level family: 189
```

Note that such information can get lost once applied `subset`, since the respective parents or children from the original data set are not anymore in the subset. May you like to recover parents and children, you can use the functions `get_paretns` or `get_children`, respectively.

```
summary(Papyrus, "all")
```

```
## ------------------------------ 
## concept ID: 206 
## view ID: 1 
## level: species 
## parent: none 
## 
## # accepted name: 
## 206 Cyperus papyrus L. 
## 
## # synonyms (2): 
## 52612 Cyperus papyrus subsp. antiquorum (Willd.) Chiov. 
## 52613 Cyperus papyrus subsp. nyassicus Chiov. 
## ------------------------------
```

```
Papyrus <- get_parents(Easplist, Papyrus)
summary(Papyrus, "all")
```

```
## ------------------------------ 
## concept ID: 206 
## view ID: 1 
## level: species 
## parent: 53660 
## 
## # accepted name: 
## 206 Cyperus papyrus L. 
## 
## # synonyms (2): 
## 52612 Cyperus papyrus subsp. antiquorum (Willd.) Chiov. 
## 52613 Cyperus papyrus subsp. nyassicus Chiov. 
## ------------------------------ 
## concept ID: 53660 
## view ID: 2 
## level: genus 
## parent: 54607 
## 
## # accepted name: 
## 53660 Cyperus NA 
## ------------------------------ 
## concept ID: 54607 
## view ID: 2 
## level: family 
## parent: none 
## 
## # accepted name: 
## 54607 Cyperaceae NA 
## ------------------------------
```

# 4. Applying taxlist to syntaxonomic schemes

## 4.1 Example of a phytosociological classification

To illustrate the flexibility of the `taxlist` objects, the next example will handle a syntaxonomical scheme. As example it will be used a scheme proposed by the author for aquatic and semi-aquatic vegetation in Tanzania (Alvarez 2017 *Phytocoenologia* in review). The scheme includes 10 associations classified into 4 classes:

## 4.2 Building the taxlist object

The content for the taxonomic list is included in a data frame and can be downloaded by following command:

```
load(url("https://github.com/kamapu/Guides/raw/master/data/wetlands_syntax.rda"))
```

The data frame `Concepts` contains the list of syntaxon names that are considered as accepted in the previous scheme. This list will be used to insert the new concepts in the `taxlist` object.

```
head(Concepts)
```

```
##   TaxonConceptID Parent                                TaxonName
## 1              1     NA                         Lemnetea minoris
## 2              2      1                 Salvinio-Eichhornietalia
## 3              3      2                       Pistion stratiotes
## 4              4      3 Lemno paucicostatae-Pistietum stratiotes
## 5              5     NA                                Potametea
## 6              6      5                       Nymphaeetalia loti
##                                   AuthorName       Level
## 1    Koch & Tüxen ex den Hartog & Segal 1964       class
## 2 Borhidi ex Borhidi, Muñiz & del Risco 1979       order
## 3                (Schmitz 1971) Schmitz 1988    alliance
## 4                                Lebrun 1947 association
## 5                Klika ex Klika & Novák 1941       class
## 6                                Lebrun 1947       order
```

```
Syntax <- new("taxlist")

levels(Syntax) <- c("association","alliance","order","class")

taxon_views(Syntax) <- data.frame(ViewID=1, Author="Alvarez M", Year=2017,
        Title="Classification of aquatic and semi-aquatic vegetation in East Africa",
        stringsAsFactors=FALSE)

Syntax <- with(Concepts, add_concept(Syntax, TaxonName=TaxonName,
                AuthorName=AuthorName, Parent=Parent, Level=Level,
                ViewID=rep(1, nrow(Concepts))))

summary(Syntax)
```

```
## object size: 10.1 Kb 
## validation of 'taxlist' object: TRUE 
## 
## number of names: 26 
## number of concepts: 26 
## trait entries: 0 
## number of trait variables: 0 
## reference entries: 1 
## 
## concepts with parents: 22 
## concepts with children: 16 
## 
## hierarchical levels: association < alliance < order < class 
## number of concepts in level association: 10
## number of concepts in level alliance: 7
## number of concepts in level order: 5
## number of concepts in level class: 4
```

Note that the function `new` created an empty object (prototype), while `levels` insert the custom levels (syntaxonomical hierarchies). For the later function, the levels have to be inserted from the lower to the higher ranks. Furthermore the reference defining the concepts included in the syntaxonomic scheme was inserted in the object using the function `taxon_views` and finally the concepts were inserted by the function `add_concept`.

The next step will be inserting those names that are considered as synonyms for the respective syntaxa. Synonyms are included in the data frame `Synonyms`.

```
head(Synonyms)
```

```
##   TaxonConceptID                             TaxonName
## 1              1                          Stratiotetea
## 2              3                  Pistion pantropicale
## 3              8               Utriculario-Nymphaeetum
## 4              8 Utriculario exoletae-Nymphaeetum loti
## 5              9                          Phragmitetea
## 6             10                           Papyretalia
##                   AuthorName
## 1    den Hartog & Segal 1964
## 2               Schmitz 1971
## 3 (Lebrun 1947) Léonard 1950
## 4    Szafranski & Apema 1983
## 5      Tüxen & Preising 1942
## 6                Lebrun 1947
```

```
Syntax <- with(Synonyms, add_synonym(Syntax, ConceptID=TaxonConceptID,
                TaxonName=TaxonName, AuthorName=AuthorName))
```

Finally, the codes provided for the associations will be inserted as traits properties) of them in the slot `taxonTraits`.

```
head(Codes)
```

```
##   TaxonConceptID Code
## 1             12  HE1
## 2             13  HE2
## 3             14  HE3
## 4             20  HE4
## 5             17  HE5
## 6             18  HE6
```

```
taxon_traits(Syntax) <- Codes
summary(Syntax)
```

```
## object size: 12.3 Kb 
## validation of 'taxlist' object: TRUE 
## 
## number of names: 37 
## number of concepts: 26 
## trait entries: 10 
## number of trait variables: 1 
## reference entries: 1 
## 
## concepts with parents: 22 
## concepts with children: 16 
## 
## hierarchical levels: association < alliance < order < class 
## number of concepts in level association: 10
## number of concepts in level alliance: 7
## number of concepts in level order: 5
## number of concepts in level class: 4
```

For instance, you may like to get the parental chain from an association ( e.g. for *Nymphaeetum loti*).

```
Nymplot <- subset(Syntax, charmatch("Nymphaeetum", TaxonName), slot="names")
summary(Nymplot, "all")
```

```
## ------------------------------ 
## concept ID: 8 
## view ID: 1 
## level: association 
## parent: none 
## 
## # accepted name: 
## 8 Nymphaeetum loti Lebrun 1947 
## 
## # synonyms (2): 
## 29 Utriculario-Nymphaeetum (Lebrun 1947) Léonard 1950 
## 30 Utriculario exoletae-Nymphaeetum loti Szafranski & Apema 1983 
## ------------------------------
```

Note that there is the logical arguments `keep_parents` and `keep_children` to preserve hierarchical information in the subset:

```
Nymplot <- subset(Syntax, charmatch("Nymphaeetum", TaxonName), slot="names",
    keep_parents=TRUE)
summary(Nymplot, "all")
```

```
## ------------------------------ 
## concept ID: 5 
## view ID: 1 
## level: class 
## parent: none 
## 
## # accepted name: 
## 5 Potametea Klika ex Klika & Novák 1941 
## ------------------------------ 
## concept ID: 6 
## view ID: 1 
## level: order 
## parent: 5 
## 
## # accepted name: 
## 6 Nymphaeetalia loti Lebrun 1947 
## ------------------------------ 
## concept ID: 7 
## view ID: 1 
## level: alliance 
## parent: 6 
## 
## # accepted name: 
## 7 Nymphaeion loti Lebrun 1947 
## ------------------------------ 
## concept ID: 8 
## view ID: 1 
## level: association 
## parent: 7 
## 
## # accepted name: 
## 8 Nymphaeetum loti Lebrun 1947 
## 
## # synonyms (2): 
## 29 Utriculario-Nymphaeetum (Lebrun 1947) Léonard 1950 
## 30 Utriculario exoletae-Nymphaeetum loti Szafranski & Apema 1983 
## ------------------------------
```

By using the function `subset` we just created a new object containing only the association *Nymphaeetum loti* and its parental chain. This subset was then used to extract the parental chain from `Syntax`.
